# Combination of sapacitabine and HDAC inhibitors stimulates cell death in AML and other tumour types

**DOI:** 10.1038/sj.bjc.6605922

**Published:** 2010-10-05

**Authors:** S R Green, A K Choudhary, I N Fleming

**Affiliations:** 1 Cyclacel Ltd., 1 James Lindsay Place, Dundee DD1 5JJ, UK

**Keywords:** sapacitabine, nucleoside analogue, histone deacetylase inhibitor, vorinostat, valproate

## Abstract

**Background:**

Alternative treatments are needed for elderly patients with acute myeloid leukaemia, as the disease prognosis is poor and the current treatment is unsuitable for many patients.

**Methods:**

In this study, we investigated whether combining the nucleoside analogue sapacitabine with histone deacetylase (HDAC) inhibitors could be an effective treatment. Synergy and mode-of-action analysis were studied in cultured cell lines and the efficacy of the combination was confirmed in a xenograft model.

**Results:**

CNDAC (1-(2-C-cyano-2-deoxy-*β*-D-arabino-pentofuranosyl)-cytosine), the active component of sapacitabine, synergised with vorinostat in cell lines derived from a range of tumour types. Synergy was not dependent on a specific sequence of drug administration and was also observed when CNDAC was combined with an alternative HDAC inhibitor, valproate. Flow cytometry and western blot analysis confirmed that the combination induced a significant increase in apoptosis. Mode-of-action analysis detected changes in Bcl-xl, Mcl-1, Noxa, Bid and Bim, which are all regulators of the apoptotic process. The sapacitabine/vorinostat combination demonstrated significant benefit compared with the single-agent treatments in an MV4-11 xenograft, in the absence of any observed toxicity.

**Conclusion:**

Sapacitabine and HDAC inhibitors are an effective drug combination that is worthy of clinical exploration.

Acute myeloid leukaemia (AML) is the most common type of acute leukaemia in adults, with ∼12 000 cases diagnosed in the United States each year (Stone *et al*, 2004). Approximately two-thirds of these patients are older than 60 years; the prognosis for elderly AML patients is particularly poor, with a median survival of ∼2 months and a 6% 2-year survival rate (Menzin *et al*, 2002). Conventional treatment for AML usually involves induction therapy comprising the combination of an anthracycline with the nucleoside analogue cytarabine. However, this treatment is very intensive and many elderly patients are considered unfit for induction treatment; accordingly there is a clear need for alternative treatments.

CNDAC (1-(2-C-cyano-2-deoxy-*β*-D-arabino-pentofuranosyl)-cytosine) is a 2′-deoxycytidine analogue that was designed specifically to have a novel DNA-strand-breaking mechanism of action (Matsuda *et al*, 1991). In contrast to other deoxycytidine analogues that block cells in the S-phase of the cell cycle (for example, gemcitabine and cytarabine), CNDAC induces an S-phase delay, followed by an arrest in G2. This effect is a function of the cyano group, which promotes the induction of single-strand (ss) DNA breaks after it has been integrated into DNA (Matsuda *et al*, 1991). These ssDNA breaks are converted into double-strand (ds) DNA breaks, which activate the dsDNA repair pathway and result in arrest at G2 (Liu *et al*, 2005). Sapacitabine (CS-682) is a palmitoyl derivative of CNDAC (Hanaoka *et al*, 1999). The fatty-acid side chain on the N4 group of the cytosine moiety improves oral bioavailability and reduces inactivation by deamination. Sapacitabine has improved efficacy over both gemcitabine and cytarabine in xenograft models (Tanaka *et al*, 1992; Hanaoka *et al*, 1999). Sapacitabine has completed phase I studies in solid tumours (Delaunoit *et al*, 2006; Gilbert *et al*, 2006) and haematological malignancies (Kantarjian *et al*, 2010) in which promising activity was reported. Sapacitabine is currently in phase II clinical trials in AML, myelodysplastic syndrome (MDS) and non-small-cell lung cancer (NSCLC).

Histone deacetylase (HDAC) inhibitors are a diverse series of compounds being developed as anti-cancer agents (Bolden *et al*, 2006). The HDAC inhibitors promote increased protein acetylation, particularly of histones. Increased acetylation has a dramatic effect on the conformation of histones and thereby the extent of DNA supercoiling; this results in changes in the cellular gene expression profile, by modulating access of transcription factors to their DNA-binding sites (Momparler, 2003; Duan *et al*, 2005). The failure of normal differentiation in haematological malignancies is often associated with poor transcription of genes involved in regulating the mature phenotype (Batty *et al*, 2009), suggesting that these diseases could be attractive targets for HDAC inhibitor treatment.

Sapacitabine has demonstrated clinical activity in a phase I study involving leukaemia patients (Kantarjian *et al*, 2010), whereas the HDAC inhibitors vorinostat and valproate have also elicited clinical responses in AML patients in phase I studies (Bug *et al*, 2005; Garcia-Manero *et al*, 2008). In the clinic, most anti-cancer drugs are used as part of a combination therapy, to enhance efficacy, reduce toxicity and decrease drug resistance. Thus the pre-clinical evaluation of the combination of these two clinically active agents would determine whether the combination could be worth clinical exploration. As CNDAC (Liu *et al*, 2005) and most HDAC inhibitors (Bolden *et al*, 2006) promote increased apoptosis through very different mechanisms, it was hypothesised that a combination of these agents might significantly stimulate cell death. This hypothesis was evaluated by combining CNDAC with HDAC inhibitors in AML cell lines and in cell lines derived from other tumours. The cellular effects of this combination were investigated in the AML cell line, MV4-11. Finally, analysis of the combination in an MV4-11 mouse xenograft model demonstrated that combination treatment significantly reduced tumour growth compared with the single-agent treatments, confirming that the combination was an effective *in vivo* therapeutic option.

## Materials and methods

### Cell lines and reagents

MV4-11, Hut78, H460 and H1299 cells were purchased from ATCC (Mannassas, VA, USA); PL21 and HL60 cells were obtained from ECACC (Porton Down, UK); and Granta-519 cells were acquired from DSMZ (Braunschweig, Germany). Cell cultures were maintained as recommended by the suppliers. All reagents were purchased from Sigma (Poole, UK) unless stated otherwise.

### Drug combination analysis

The cell lines were seeded in 96-well plates at the following cell densities: Granta-519, 25 000 cells per well; MV4-11, PL21 and Hut78, 8000 cells per well; HL60, 5000 cells per well; H460, 3000 cells per well; and H1299, 2000 cells per well. Stock solutions of CNDAC (Cyclacel Ltd., Dundee, UK) and vorinostat (Toronto Research Chemicals, North York, Canada) were prepared in dimethylsulphoxide. Valproate was dissolved in 0.9% (wt/vol) saline.

Three treatment regimens were explored (CNDAC pre-treatment, HDAC inhibitor pre-treatment and concomitant treatment). In most cell lines, a 72-h concomitant treatment regime was employed. This involved simultaneous treatment of cells with CNDAC and either vorinostat or valproate, alongside suitable controls of cells treated with the individual compounds alone. A 48-h concomitant treatment regime was used in the AML cell lines.

In the adherent cell lines (H460 and H1299), sequential treatment regimes involved adding one drug to the cells 2 h after plating, and leaving for a 24-h period. Media was then aspirated and replaced with fresh media containing the second drug, which was left for a further 72 h. The two individual treatment controls for the sequential treatment regime involved substituting one of the drug treatments with drug-free media. A similar sequential protocol was employed in the suspension cell lines, except that medium was not aspirated from the cells after the 24-h pre-treatment. Therefore, the concentration of the pre-treatment drug was reduced on addition of fresh medium containing the second agent. In addition, a 48-h treatment time was used for the second drug treatment in the AML cell lines.

After the drug treatments were completed, the number of viable cells in each well was estimated by incubating in media containing 10% alamar blue (Roche, Lewes, UK) and measuring the absorbance at 488–595 nm. Drug interactions were analysed using the Calcusyn software (BioSoft, Cambridge, UK). A combination index (CI) value of 1 indicated an additive drug interaction, whereas a CI value >1 suggested antagonism and a value <1 denoted synergism.

### Western blot analysis

MV4-11 and HL60 cells were seeded in 10-cm plates at 9 × 10^5^ cells per plate, Hut78 cells at 1.4 × 10^6^ cells per plate and H460 cells at 6 × 10^5^ cells per plate. Cells were treated with various concentrations of CNDAC, vorinostat or a combination of these agents. After incubation, cells were harvested by centrifugation at 2000 r.p.m. for 5 min, washed once with ice-cold buffer A (50 mM HEPES (pH 7.0), containing 20 mM NaCl, 1 mM DTT, 10 mM sodium pyrophosphate, 10 mM sodium fluoride, 1 mM sodium orthovanadate and protease inhibitors) and then resuspended in 150 *μ*l of buffer A. All samples were lysed by sonication. Lysates (10 *μ*g protein per well) were resolved on 12% acrylamide bis–tris gels (Invitrogen, Paisley, UK) and the proteins transferred to nitrocellulose membranes (Schleicher & Schuell, Dassel, Germany).

Membranes were blocked for 1 h at room temperature in phosphate-buffered saline containing 0.02% (vol/vol) Tween 20 (PBST) and 5% (wt/vol) fat-free dried milk. Membranes were probed with the following primary antibodies: Noxa (Calbiochem, Nottingham, UK), Mcl-1 and Bcl-xl (Santa Cruz Biotechnology, Santa Cruz, CA, USA), survivin (Abcam, Cambridge, UK), histone H2AX, phosphoserine 139 histone H2AX, acetyl histone H4, Bcl-2, Bax and Bim (Millipore, Watford, UK), XIAP and Asp-214 cleaved PARP (poly-ADP ribose polymerase; BD Pharmingen, Oxford, UK), PUMA, Bid and cleaved caspase 3 (Cell Signalling, Hitchin, UK) and p53 (Oncogene, Nottingham, UK). Primary antibody incubations were carried out overnight at 4 °C in PBST containing 3% (wt/vol) dried milk, with the exception of PUMA, which was incubated in PBST containing 2% (wt/vol) bovine serum albumin. Membranes were washed three times in PBST, and then incubated for 1 h with the appropriate horseradish-peroxidase-conjugated secondary antibody (Pierce, Cramlington, UK) diluted in the ratio 1 : 6000 in PBST containing 3% (wt/vol) dried milk. Membranes were washed three times in PBST before antibody detection using ECL reagent (Amersham Corp., Little Chalfont, UK) or Immobilon HRP substrate (Millipore).

### Flow cytometry assays

The cell cycle profile was analysed by propidium iodide staining of ethanol-fixed cells as described previously (Fleming *et al*, 2008). Cells were seeded and treated as for western blots.

The Bax/Bak activation assay was performed as described previously (Willis *et al*, 2005). Antibodies used in this experiment were: activated Bax (6A7, Santa Cruz Biotechnology), activated Bak (Ab1; Calbiochem) and fluorescein isothiocyanate (FITC)-conjugated mouse secondary antibody (Abcam).

### *In vivo* studies

Female (*nu/nu*) mice were injected subcutaneously with 1 × 10^7^ MV4-11 cells resuspended in 50% Matrigel (BD Biosciences) at a single site on their flanks. Once tumour volumes were 126–256 mm^3^ (16 days post-implantation) animals were pair matched by tumour size into treatment groups (minimum of six mice per group) with a mean tumour size of ∼190 mm^3^. Tumour measurements were calculated using the formula: volume (mm^3^)=width^2^ (mm) × length (mm) × 0.5. Sapacitabine (Cyclacel Ltd.) was prepared in 2.5% dimethylacetate and 9.75% emulphor (Alkamuls EL-620, Rhodia, Cranbury, NJ, USA), whereas vorinostat was prepared in 40% hydroxypropyl-*β*-cyclodextrin vehicle. Sapacitabine was administered once a day orally (5 or 15 mg kg^−1^) for 4 days, followed by a 3-day break before another 4 days of treatment; dosing started on the same day as distribution to the treatment groups. Vorinostat (33 mg kg^−1^) was administered by intraperitoneal injection once a day for 12 consecutive days starting on the day after the mice were randomised. The groups treated with the combination were dosed in the same manner as both the single-agent groups. Mice were weighed daily for 5 days of treatment and then at least twice a week to assess toxicity. No treatment-related deaths were seen in this experiment. The tumours were measured at least twice a week to determine tumour volume. The percent tumour growth inhibition (TGI) was determined using the formula: 1−(mean change in treated tumour volume/mean change in control tumour volume) × 100. Statistical significance for the experiment was determined using a one-way analysis of variance (ANOVA) test. Significance between different treatment groups was determined using a two-sided unpaired Student's *t*-test. Xenografts were performed at the Piedmont Research Centre. Studies were reviewed by an internal board to confirm that procedures complied with the recommendations of the *Guide for Care and Use of Laboratory Animals*.

## Results

### *In vitro* drug combination analysis of CNDAC and HDAC inhibitors

The aim of this series of experiments was to determine whether combining CNDAC with HDAC inhibitors would produce a synergistic drug interaction. Initial evaluation of the combination was carried out in the AML cell lines MV4-11, HL60 and PL21. Combining CNDAC with either vorinostat (Table 1A) or valproate (Table 1B) generated CI values that represented predominantly either synergistic or additive drug interactions. Three treatment regimens were explored (CNDAC pre-treatment, HDAC inhibitor pre-treatment and concomitant treatment); CNDAC treatment before vorinostat generated slightly stronger synergy (lower CI values) than the other schedules tested. The CNDAC/valproate combination generated similar results with all three schedules tested.

These findings were expanded to include cell lines derived from other tumour types, including H460 and H1299 (NSCLC), Hut78 (cutaneous T-cell lymphoma) and Granta-519 (non-Hodgkin's lymphoma). The CNDAC/vorinostat combination predominantly generated synergy in Hut78, Granta-519 and H1299 cells, whereas it produced a mostly additive drug interaction in H460 cells (Table 1A). Similarly, combination of CNDAC and valproate produced synergy in H1299 cells and weak synergy/additivity in H460 cells (Table 1B).

Overall, this series of results demonstrated that combining CNDAC with an HDAC inhibitor produced a synergistic increase in cytotoxicity in cell lines derived from diverse tumour types. Synergy was observed in cell types that contain either wild-type p53 (MV4-11, H460 and Granta-519) or mutant p53 (HL60, H1299 and Hut78), suggesting that the p53 status was not a critical factor for the combination. Moreover, combining CNDAC with either vorinostat or valproate produced broadly similar CI values in these cell lines, suggesting that the observed synergy was a class effect, and that the selection of the HDAC inhibitor did not appear to be particularly crucial.

### The combination of CNDAC and vorinostat induced cell death in MV4-11 cells

**Figure 1:**
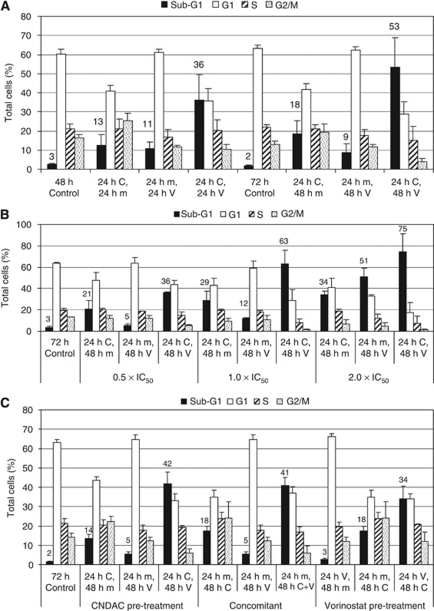
Flow cytometry analysis of the CNDAC/vorinostat combination in MV4-11 cells. MV4-11 cells were treated with dimethylsulphoxide control (m), CNDAC (C), vorinostat (V) or combinations of these agents (CV), as indicated. (**A**) Cells were treated for the indicated time periods using a schedule involving 24-h CNDAC pre-treatment followed by vorinostat (for either 24 or 48 h). Compounds were used at 1 × IC_50_. The cell cycle profile was determined after the total treatment time periods of either 48 or 72 h. (**B**) The cell cycle profile of MV4-11 cells was analysed after treatment with various concentrations of compound, using a schedule involving 24-h CNDAC pre-treatment followed by 48-h vorinostat. (**C**) The cell cycle profile was determined after cells had been treated with different treatment regimes: CNDAC pre-treatment, concomitant treatment and vorinostat pre-treatment. Compounds were used at 1 × IC_50_. Results are given as the average ± s.d. of three independent experiments, except in panel **B**, which is the average ± s.d. of duplicate experiments. CNDAC IC_50_=0.46 *μ*M; vorinostat IC_50_=0.4 *μ*M.

MV4-11 cells were used for more in-depth analysis of the combination, as this well-established AML model can be used both *in vitro* and *in vivo*. To evaluate the cellular effects of the combination in more detail, MV4-11 cell cycle profiles were analysed by flow cytometry following treatment with compounds at concentrations equivalent to their IC_50_ value (Figure 1A). A CNDAC pre-treatment regimen was explored because this was the most synergistic combination in the original cytotoxicity experiments (Table 1A). Under the conditions tested, both the CNDAC (C) (24 h C, 24 h m (media); 24 h C, 48 h m) and vorinostat (V) (24 h m, 24 h V; 24 h m, 48 h V) single-agent treatments caused a small increase in the number of cells in the sub-G1 cell population (cells with a DNA content lower than normal diploid cells), which is indicative of dead cells. On the other hand, the combination of CNDAC followed by vorinostat (24 h C, 24 h V; 24 h C, 48 h V) resulted in a dramatic increase in the sub-G1 population indicating a synergistic increase in cell death at both the 48- and 72-h time points. The total treatment time of 72 h resulted in a greater number of dead cells (53%) than the 48-h total treatment time (36%). To optimise the synergistic interaction between the two agents, different concentrations of drug (corresponding to 0.5 × , 1 × and 2 × IC_50_) were evaluated during a 72-h incubation (Figure 1B). CNDAC and vorinostat single-agent treatments induced a dose-dependent increase in the sub-G1 cell population. Combination of CNDAC with vorinostat induced a significant increase in the sub-G1 cell population, compared with the single-agent treatments; this effect was also dose dependent. The induction of cell death was clearly synergistic when the compounds were used at either 0.5 × or 1 × IC_50_, but only appeared additive when used at 2 × IC_50_; the apparent lack of synergy at 2 × IC_50_ was presumably because of the fact that both single-agent treatments induced such a significant proportion of cell death on their own, making it difficult to evaluate synergy. Finally, in this initial set of experiments, MV4-11 cells were treated with the CNDAC/vorinostat combination using the three treatment schedules evaluated in the cytotoxicity experiments to ascertain whether the sequence of compound administration had a significant influence on the amount of cell death. The results (Figure 1C) demonstrated that a synergistic increase in the sub-G1 cell population was seen for all three combination treatment schedules, but the CNDAC pre-treatment (42%) and the concomitant treatment (41%) induced a slightly higher proportion of cell death than the vorinostat pre-treatment (34%). This data correlated well with the CI values detected in the cytotoxicity experiments (Table 1A). These results indicated that the sequence of administration was not critical for the combination, and suggested that the synergy was not the result of one treatment blocking the cells in a specific phase of the cell cycle where the second treatment was more effective.

### Mode-of-action analysis of the CNDAC/vorinostat combinations in MV4-11 cells

**Figure 2:**
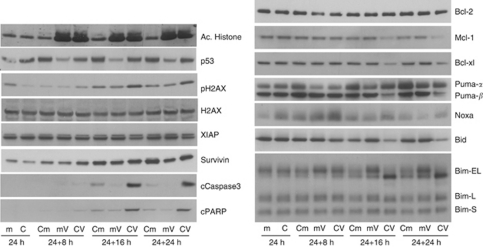
Combination of CNDAC and vorinostat induces selective modulation of apoptotic proteins in MV4-11 cells. MV4-11 cells were treated with dimethylsulphoxide (m), 1 × IC_50_ CNDAC (C), 1 × IC_50_ vorinostat (V), or 1 × IC_50_ CNDAC and vorinostat (CV). The schedule involved 24-h CNDAC pre-treatment followed by vorinostat. Cells were harvested at 0, 8, 16 or 24 h after addition of vorinostat, as indicated. The resulting lysates (10 *μ*g) were resolved on 12% acrylamide bis–tris gels, transferred to nitrocellulose membranes and probed with the antibodies shown. cPARP, cleaved PARP; cCaspase 3, cleaved caspase 3. The lower band in the XIAP blot represents XIAP, whereas the upper band is an unknown protein. Results are representative of two independent experiments.

Western blot analysis was used to study the molecular changes that were induced by the CNDAC/vorinostat combination (Figure 2). Cells were initially treated with either CNDAC (C) or media (m) for 24 h before an equivalent volume of media being added, which was either drug free (m) or contained vorinostat (V), and the incubation continued for a further 8–24 h. As expected, vorinostat stimulated an increase in histone acetylation. Similarly, CNDAC enhanced p53 levels in MV4-11 cells, a property that has been established for cytarabine (Kobayashi *et al*, 1998) and gemcitabine (Achanta *et al*, 2001). The combination stimulated a modest increase in histone H2AX phosphorylation at serine 139, over that seen by either single agent. Phospho-H2AX is a marker of dsDNA damage, but as this effect was relatively minor, it suggested that enhancement of DNA damage was not the main cause of the synergistic increase in cell death. The combination induced a synergistic time-dependent increase in cleaved PARP, detectable ∼16 h after adding vorinostat to CNDAC-treated cells (Figure 2). A synergistic increase in the active cleaved form of caspase 3, an executioner caspase, became evident at the same time as the cleaved PARP. These two observations confirmed that the increase in cells with a sub-G1 DNA content (Figure 1) was because of the induction of apoptosis.

Apoptosis is regulated by the Bcl-2 family proteins and the related BH3-only proteins. These proteins can be divided into two groups, with either pro- or anti-apoptotic roles; modifying the levels or activity of these proteins disrupts the cellular apoptotic balance. Western blots confirmed that treatment with the CNDAC/vorinostat combination reduced the levels of the anti-apoptotic proteins Mcl-1 and Bcl-xl to a much greater degree than either individual compound, but had no significant effect on Bcl-2 (Figure 2). Overall, the loss of Mcl-1 and Bcl-xl would push the cells towards apoptosis. The BH3-only proteins are a family of small pro-apoptotic proteins that connect various stress stimuli with the Bcl-2 proteins (Shibue and Taniguchi, 2006). Western blot analysis was used to study the effect of combination treatment on Noxa and PUMA, as CNDAC upregulated p53 protein levels (Figure 2) and these proteins are transcriptionally activated by p53 (Shibue and Taniguchi, 2006). CNDAC treatment induced both Noxa and PUMA*α*, whereas the combination induced a slightly greater transient increase in Noxa, but had little effect on the levels of PUMA*α* (Figure 2). The BH3-only protein Bid has an important role in apoptosis through a mechanism that involves its own degradation (Shibue and Taniguchi, 2006). CNDAC treatment caused a time-dependent decrease in the level of full-length Bid, which was enhanced by the combination with vorinostat (Figure 2), suggesting that the induction of cell death involved the Bid pathway. Another BH3-only protein, Bim, has three isoforms: extra-long (EL), long (L) and short (S). Bim-S is constitutively active, whereas Bim-EL and -L are regulated by reversible phosphorylation (Wang *et al*, 2004; Shibue and Taniguchi, 2006). All three isoforms of Bim were present in MV4-11 cells, and Bim-E and -EL appeared to be composed of multiple bands. Both vorinostat and the combination treatment enhanced the levels of Bim-EL, consistent with previous vorinostat studies (Fandy *et al*, 2005; Zhao *et al*, 2005). Combination treatment also increased the electrophoretic mobility of Bim-EL (Figure 2). Such a mobility shift is consistent with a change in a protein's phosphorylation state; several studies have reported that dephosphorylation of Bim-EL increases its electrophoretic mobility and stimulates apoptosis by allowing Bim-EL to directly bind to and activate Bax (Harada *et al*, 2004; Wang *et al*, 2004). The observed changes in the BH3-only proteins Noxa, Bid and Bim occurred before the increase in cleaved PARP, suggesting that they contributed towards the increased apoptosis. In contrast, the CNDAC/vorinostat combination did not induce dephosphorylation of Bad (data not shown), suggesting that the combination does not activate the Bad pro-apoptotic pathway. The combination also promoted a modest downregulation of the caspase inhibitor XIAP (Figure 2), which would also contribute to enhanced apoptosis.

**Figure 3:**
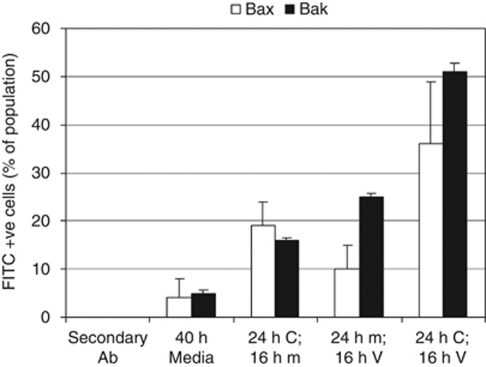
The CNDAC/vorinostat combination activates Bax and Bak in MV4-11 cells. MV4-11 cells were treated with dimethylsulphoxide (m), 1 × IC_50_ CNDAC (C), vorinostat (V) or both compounds in a sequential manner (CV). Combination treatment involved 24-h CNDAC pre-treatment followed by 16-h vorinostat treatment. One of the drug treatments was replaced by a drug-free media treatment in the two single-agent controls (m). Cells were then harvested and the resulting lysates used to measure the levels of activated Bax or Bak. Some of the samples were incubated with secondary antibody in the absence of primary antibody (secondary Ab) to assess non-specific binding of the FITC secondary antibody. Results are the average ± s.d. of two independent experiments.

The pro-apoptotic Bcl-2 family proteins Bax and Bak are the key drivers of apoptosis; when activated, these proteins permeabilize the outer mitochondrial membrane and promote the release of pro-apoptotic factors (for example, cytochrome *c*) that activate caspases and initiate the apoptotic cascade. Western blot analysis demonstrated that the combination did not increase the expression of either of these proteins (data not shown); hence, a flow cytometry-based assay was performed to investigate whether the observed increase in apoptotic cells involved activation of Bax or Bak. When either Bax or Bak become activated, they undergo a conformational change, which reveals a neo-epitope that is recognised by specific antibodies. The antibodies are labelled with the FITC dye; hence, cells with increased FITC signal contain activated Bax or Bak. The results indicated that combining CNDAC and vorinostat promoted activation of both Bax and Bak in MV4-11 cells (Figure 3), and that the combination was slightly more effective at activating Bak than Bax. As activation of Bax or Bak reflects a commitment towards apoptotic cell death, these data provide further evidence that the combination stimulated apoptosis in MV4-11 cells.

### The combination of CNDAC and vorinostat can induce apoptosis in cell lines derived from different tumour types

**Figure 4:**
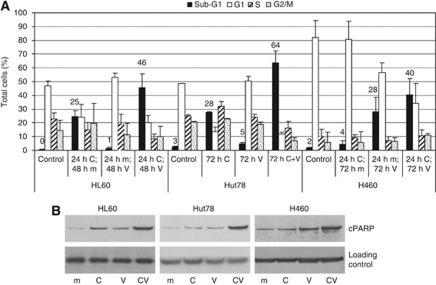
The CNDAC/vorinostat combination stimulates apoptosis in cell lines derived from various tumour types. HL60, Hut78 and H460 cells were treated with 1 × IC_50_ CNDAC, vorinostat or both compounds. (**A**) Cell cycle profile of each cell line was analysed after propidium iodide staining. Treatment schedules were: HL60 cells, 24-h CNDAC pre-treatment followed by 48-h vorinostat treatment; Hut78 cells, 72-h concomitant treatment; H460 cells, 24-h CNDAC pre-treatment followed by 72-h vorinostat treatment. Results are the average of three independent experiments. (**B**) Cells were treated with dimethylsulphoxide (m), 1 × IC_50_ CNDAC (C), 1 × IC_50_ vorinostat (V) or 1 × IC_50_ CNDAC and vorinostat (CV). Treatment schedules were: HL60 cells, 24-h CNDAC pre-treatment followed by 16-h vorinostat treatment; Hut78 cells, 32-h concomitant treatment; H460 cells 24-h CNDAC pre-treatment followed by 48-h vorinostat treatment. The resulting lysates (20 *μ*g) were resolved on 12% acrylamide bis–tris gels, transferred to nitrocellulose membranes and probed with cPARP antibody or an appropriate loading control (histone H2AX in HL60 and Hut78, actin in H460). Results are representative of two independent experiments. In HL60 cells, the IC_50_ values for CNDAC and vorinostat were 0.14 and 0.68 *μ*M, respectively. In Hut78 cells, the IC_50_ values for CNDAC and vorinostat were 3.6 and 0.46 *μ*M, respectively. In H460 cells, the IC_50_ values for CNDAC and vorinostat were 0.25 and 2.5 *μ*M, respectively.

As combining CNDAC with vorinostat produced a significant increase in the amount of apoptosis in MV4-11 cells (Figure 1), these studies were expanded to ascertain whether the observed cytotoxic synergy detected in other cell lines (Table 1) also involved the induction of apoptosis. HL60, Hut78 and H460 cells were chosen for further study, as they are from diverse tumour types and exhibited a range of CI values with the CNDAC/vorinostat combination. Based on the cytotoxicity data (Table 1) an appropriate treatment regimen was selected for each cell line. Cell cycle analysis demonstrated that combining CNDAC with vorinostat significantly increased the population of cells with a sub-G1 DNA content in HL60 and Hut78 cells and induced a modest sub-G1 increase in H460 cells (Figure 4A). A similar trend was observed by analysing the levels of cleaved PARP; combination treatment induced a significant increase in PARP cleavage in HL60 and Hut78 cells and a moderate increase in H460 cells, compared with the single-agent treatments (Figure 4B). These results were in general agreement with the combination analysis data and confirm that this combination appeared to be effective in cell lines derived from multiple tumour types.

### *In vivo* evaluation of sapacitabine and vorinostat in combination

**Figure 5:**
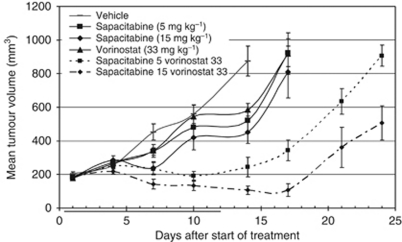
The effect of the combination of sapacitabine and vorinostat on the growth of an MV4-11 xenograft. Mice (*nu/nu*) were injected subcutaneously with ∼1 × 10^7^ MV4-11 cells per mouse at a single site on their flanks. Treatment was initiated when tumours were within the range 126–256 mm^3^ and was continued for up to 12 days. Results show mean tumour volume (±s.e.m.) for each treatment group (⩾6 mice per group) and represent vehicle, sapacitabine (5 or 15 mg kg^−1^), as a once-daily oral treatment for 4 consecutive days followed by a 3-day break and then a further 4 days of treatment, vorinostat (33 mg kg^−1^) daily by intraperitoneal injection for 12 consecutive days or the combination of both agents.

To determine whether these *in vitro* results translated into the *in vivo* setting, the combination was evaluated in an MV4-11 xenograft (Figure 5). The palmitoyl derivative CNDAC prodrug, sapacitabine, was used *in vivo* to maximise oral bioavailability. There were no treatment-related deaths during the experiment, suggesting that there was no adverse toxicity associated with the combination. In the control group, some mice were killed due to tumour burden (>1000 mm^3^) on day 14; hence, this was considered the experimental end point for statistical analysis. On day 14, the single-agent treatments appeared to have a reduced mean tumour size compared with the vehicle control (875 mm^3^). However, at the low doses evaluated in this experiment, the impact on tumour growth was relatively minimal, with all three single-agent treatment groups reaching the experimental end point by day 17. Unlike the single-agent treatments, combination of vorinostat with sapacitabine provided clear benefit throughout the course of the experiment. On Day 14, the sapacitabine (5 mg kg^−1^) + vorinostat (33 mg kg^−1^) group had a mean tumour volume of 245 mm^3^ and a TGI of 92%, whereas the sapacitabine (15 mg kg^−1^) + vorinostat (33 mg kg^−1^) group had a mean tumour volume of 107 mm^3^ and a TGI of 112%. An unpaired Student's *t*-test demonstrated that both combination treatments were significantly different to the appropriate controls, which resulted in an overall highly significant one-way ANOVA test (*P*<0.00001). These data demonstrate that there was a clear benefit of combining sapacitabine with vorinostat in this AML tumour model and that the combination was effective *in vivo*.

## Discussion

Conventional treatment for AML involves combination of an anthracycline with the nucleoside analogue cytarabine. However, the poor prognosis for AML patients in general, and elderly patients in particular (Menzin *et al*, 2002), means that alternative therapies are required. In this report, we investigated whether combining the novel nucleoside analogue CNDAC with HDAC inhibitors could be an effective treatment option. The resulting data provide compelling evidence that combining CNDAC with HDAC inhibitors produces an effective cytotoxic combination, both *in vitro* and *in vivo*. Moreover synergy was observed by combining CNDAC with either vorinostat or valproate, suggesting that there is a class effect between CNDAC and HDAC inhibitors.

Two distinct mechanisms can contribute towards synergy: inhibition of cell growth or induction of cell death. Evidence from multiple approaches demonstrated that the CNDAC/vorinostat combination stimulated a large increase in cell death, suggesting that this was making a significant contribution to the observed synergy. First, cell cycle analysis demonstrated that combination treatment induced a synergistic increase in cells with a sub-G1 DNA content (Figures 1 and 4); combining these agents killed approximately 50–70% of the MV4-11 cell population when treated at 1 × IC_50_ for 72 h (Figure 1A and B). Second, the combination stimulated the conformational changes associated with activation of the pro-apoptotic proteins Bax and Bak (Figure 3), and increased the level of cleaved caspase 3 and cleaved PARP (Figure 2), providing clear evidence of an increase in apoptosis. Third, the data demonstrated that CNDAC synergised with HDAC inhibitors in diverse cell lines derived from both haematological malignancies and solid tumours (Table 1, Figures 1 and 4). These data demonstrate that the combination is effective in a range of different tumour types and that the efficacy of the combination does not depend on a precise genetic signature.

Previous studies have investigated whether combining HDAC inhibitors with nucleoside analogues stimulated apoptosis. Valproate enhanced the percentage of annexin V-positive cells induced by cytarabine treatment in cultured AML cells (Siitonen *et al*, 2005). Similarly, combining vorinostat with gemcitabine stimulated apoptosis in NSCLC (Rundall *et al*, 2005) and pancreatic cancer (Arnold *et al*, 2007) cell lines. None of these earlier combination studies delineated a molecular mechanism that could account for the observed increase in apoptosis. A recent review proposed several different mechanisms to explain synergy between HDAC inhibitors and other chemotherapeutic agents (Frew *et al*, 2009). The simplest hypothesis was that pre-treatment with an HDAC inhibitor increased the accessibility of DNA-damaging agents to their target by altering the chromatin structure, thus enhancing the cytotoxic effects. This hypothesis alone could not account for the synergy between CNDAC and vorinostat, as there was no schedule dependency for the combination (Figure 1C). A second hypothesis implied that HDAC inhibitors could synergise with DNA-damaging agents, either by inducing DNA damage themselves or suppressing the DNA repair process. It has been demonstrated that HDAC inhibitor treatment can induce rapid acetylation of histone proteins and phosphorylation of histone H2AX, followed by the appearance of cleaved PARP several hours later (Gaymes *et al*, 2006). In the data reported here, the combination of CNDAC with vorinostat only induced a modest increase in H2AX phosphorylation (Figure 2), which became evident at the same time as the cleaved PARP. These data suggest that the increase in H2AX phosphorylation was an early marker of cell death rather than an apoptotic stimulus, indicating that the synergy was not a result of the HDAC inhibitor contributing to effects on DNA damage/repair. A third potential mechanism behind the synergy was that HDAC inhibitors could sensitise cells to the apoptotic effect of cytotoxic drugs by combining to decrease the cellular apoptotic threshold, through altered expression of key apoptotic regulators (Frew *et al*, 2009). As discussed below, the data presented herein are consistent with this hypothesis.

Bak and Bax are the key drivers of apoptosis. Active Bax and Bak permeabilize the mitochondrial outer membrane, releasing pro-apoptotic proteins such as cytochrome *c* and Smac/DIABLO into the cytosol, where they initiate and promote caspase activation. The Bak and Bax activity is regulated by a complex interplay between the anti-apoptotic Bcl-2 family members and the pro-apoptotic BH3-only proteins. Recent studies have revealed that the Bcl-2 family proteins only bind certain BH3-only proteins (Shibue and Taniguchi, 2006): Bcl-2 interacts with Bad, Bim or PUMA; Bcl-xl binds Bad, Bid, Bim or PUMA; and Mcl-1 is regulated by Bim, PUMA or Noxa. The combination of CNDAC and vorinostat produced discernable changes in the levels of a number of the proteins that regulate apoptosis, including decreases in the anti-apoptotic proteins Mcl-1 and Bcl-xl, and increases in the levels or activation state of the pro-apoptotic BH3-only proteins Noxa, Bid and Bim-EL (Figure 2). Modest molecular changes in all of these proteins were caused by CNDAC and/or vorinostat single-agent treatments, which suggested that the observed synergy was probably a result of the concerted action of the two agents on the apoptotic proteins. These changes would significantly disrupt the interplay between the apoptotic regulatory proteins causing the resultant activation of Bak and Bax and the induction of apoptosis.

Recent combination studies have suggested that targeting multiple arms of the apoptotic regulatory machinery is an effective strategy for killing cells (Chen *et al*, 2007; Rosato *et al*, 2007). Drug combinations that target Mcl-1 and Bcl-2/Bcl-xl may mimic the actions of more physiological regulators of apoptosis (Dai and Grant, 2007), and selective targeting of multiple apoptotic pathways may overcome functional redundancy and force cells into apoptosis. The *in vitro* studies presented here are consistent with this hypothesis; the CNDAC/vorinostat combination (Table 1 and Figure 1) targets multiple arms of the apoptotic machinery, producing changes in the levels of key proteins involved in regulating apoptosis (Figures 2, 3 and 4).

Based on the promising *in vitro* data, the studies were expanded into an *in vivo* MV4-11 xenograft model. The doses of both agents were significantly below the previously reported maximum tolerated doses (Hanaoka *et al*, 1999; Butler *et al*, 2000) and, as would be expected, showed minimal efficacy when used as single agents (Figure 5). However, the combination resulted in significantly improved efficacy, including regressions in a number of tumours at the higher dose levels tested. Moreover, the relatively low doses of drug used meant that there was no associated toxicity or weight loss in either of the single-agent arms or the combination groups. Maximum mean weight loss for a group was <4%. Although there was tumour re-growth once the dosing ceased, treatment could have been continued for additional cycles, as the agents were so well tolerated. The results of the MV4-11 xenograft confirmed that the promising *in vitro* synergy translated into the *in vivo* setting.

Owing to the limitations of the available therapy, there is an urgent need for new therapeutic options for AML patients, particularly those aged ⩾70. Currently, both sapacitabine and vorinostat are being evaluated in clinical studies, and have reported single-agent activity in phase I studies in AML and MDS patients (Garcia-Manero *et al*, 2008; Kantarjian *et al*, 2010). Sapacitabine is being explored because of the well-established activity of cytosine analogues such as cytarabine in myeloid diseases. The HDAC inhibitors may be a good option, because in myeloid malignancies, failure of normal differentiation often results from an inability to transcribe genes that encode proteins that either mediate or define the mature phenotype (Batty *et al*, 2009). For optimal transcription of these genes, histones should be in a maximally acetylated state; hence, HDAC inhibitors could provide a good therapeutic strategy for treating AML and other haematological malignancies. The data reported herein suggest that exploring the combination of sapacitabine and vorinostat in the clinic would be a valid option, especially if they could be used at reduced doses while maintaining improved efficacy.

## Figures and Tables

**Table 1:** Summary of combination studies involving CNDAC and either vorinostat or valproate

	**CNDAC pre-treatment**			**Vorinostat pre-treatment**			**Concomitant**		
**Cell line**	**ED_50_**	**ED_75_**	**ED_90_**	**ED_50_**	**ED_75_**	**ED_90_**	**ED_50_**	**ED_75_**	**ED_90_**
*(A)*									
MV4-11	1.19	0.86	0.68	0.87	0.77	0.73	1.04	0.86	0.77
HL60	1.18	0.76	0.53	1.31	0.97	0.89	1.20	0.97	0.98
PL21	0.99	0.71	0.53	1.12	0.87	0.70	1.29	0.97	0.73
Hut78	0.66	0.56	0.48	0.64	0.47	0.36	0.99	0.70	0.51
Granta-519	0.82	0.51	0.37	0.86	0.88	1.06	0.72	0.73	0.76
H460	0.93	0.91	0.94	1.63	1.58	1.57	0.85	0.99	1.18
H1299	2.24	0.42	0.60	0.66	0.84	1.90	0.63	0.59	0.99
									
	**CNDAC pre-treatment**			**Valproate pre-treatment**			**Concomitant**		
**Cell line**	**ED_50_**	**ED_75_**	**ED_90_**	**ED_50_**	**ED_75_**	**ED_90_**	**ED_50_**	**ED_75_**	**ED_90_**
*(B)*									
MV4-11	1.34	0.86	0.61	1.06	0.79	0.64	0.69	0.64	0.64
HL60	1.93	1.27	0.91	1.43	1.08	0.98	1.16	0.83	0.77
PL21	1.05	0.85	0.79	1.26	0.96	0.81	1.68	1.16	0.89
H460	1.13	0.98	0.86	1.34	1.16	1.01	1.59	1.30	1.10
H1299	1.01	0.93	0.88	0.71	0.59	0.65	0.69	0.56	0.62

Abbreviations: CNDAC=1-(2-C-cyano-2-deoxy-*β*-D-arabino-pentofuranosyl)-cytosine; ED=effective dose.

CNDAC was tested in combination with either vorinostat (A) or valproate (B) in various cell lines, using the protocol described in Materials and Methods. Concomitant and sequential treatment schedules were tested, and the resulting combination index (CI) values shown for ED_50_, ED_75_ and ED_90_ (the points on the curve where cellular proliferation is inhibited by 50, 75 and 90%, respectively). Results are the average of at least three independent experiments. The CI value definitions are as follows: 1.45–1.2 is moderately antagonistic, 1.2–1.1 is slightly antagonistic, 1.1–0.9 is additive, 0.9–0.85 is slightly synergistic, 0.85–0.7 is moderately synergistic and 0.7–0.3 is synergistic.
